# Prevalence, Trends, and Outcomes of Pulmonary Embolism Treated with Mechanical and Surgical Thrombectomy from a Nationwide Inpatient Sample

**DOI:** 10.3390/clinpract12020024

**Published:** 2022-03-13

**Authors:** Shalini Raghupathy, Achala Prashant Barigidad, Raydiene Doorgen, Shrestha Adak, Rohma Rafique Malik, Gaurav Parulekar, Jeet Janak Patel, Santh Prakash Lanka, George Mohan Varghese, Mohammed Rashid, Urvish Patel, Achint Patel, Ya-Ching Hsieh

**Affiliations:** 1 Department of Surgery, K.A.P. Vishwanadham Government Medical College, Trichy 620001, Tamil Nadu, India; dr.raghupathy.shalini@gmail.com; 2 Department of Surgery, Bangalore Medical College and Research Institute, Bengaluru 560002, Karnataka, India; achala.prashant@gmail.com; 3 Department of Surgery, American University of Antigua, St. John’s P.O. Box W1451, Coolidge, Antigua and Barbuda; raydiene.doorgen@gmail.com; 4 Department of Surgery, Kolkata Medical College and Hospital, Kolkata 700073, West Bengal, India; dr.shrestha.adak@gmail.com; 5 Department of Anesthesia, Ras Al Khaimah College of Medical Sciences, Ras Al Khaimah P.O. Box 11172, United Arab Emirates; dr.rohma.malik@gmail.com; 6 Department of Biology, York University, Toronto, ON M3J 1P3, Canada; par.gaurav13@gmail.com; 7 Department of Surgery, B.J. Medical College, Ahmedabad 380016, Gujarat, India; dr.jeetjanakpatel@gmail.com; 8 Department of Surgery, Rangaraya Medical College, Kakinada 533001, Andhra Pradesh, India; santhprakash90@gmail.com; 9 Department of Medicine, Kasturba Medical College, Manipal 576104, Karnataka, India; georgemohanvarghese@gmail.com; 10 Department of General Surgery, University of Illinois Metropolitan Group Hospitals, Chicago, IL 60657, USA; mohammad.yousif.rer@gmail.com; 11 Department of Public Health, Icahn School of Medicine Mount Sinai, New York, NY 10029, USA; dr.urvish.patel@gmail.com (U.P.); drachintpatel@gmail.com (A.P.)

**Keywords:** pulmonary embolism, surgical thrombectomy, mechanical thrombectomy, national inpatient sample

## Abstract

Pulmonary embolism (PE) is the third most common vascular disease in the US, a frequently underdiagnosed and potentially fatal condition where embolic material blocks one or more pulmonary arteries impairing blood flow. In this study, we aim to describe the prevalence, outcomes, and predictors of mortality of PE patients treated with mechanical (MT) and surgical thrombectomy (ST). This is a retrospective study using the Agency for Healthcare Research and Quality’s HCUP NIS data from 2010–2018. We used the ninth and tenth revisions of the International Classification of Diseases clinical modification codes (ICD-9-CM and ICD-10-CM) to identify patients admitted with a primary diagnosis of PE (ICD-10-CM codes I26.02, I26.09, I26.92, I26.93, I26.94, and I26.99; ICD-9-CM codes 415.11, 415.13, and 415.19). We extracted demographics, hospital-level, and patient-level characteristics, and defined the severity of comorbid conditions using Deyo modification of the Elixhauser Comorbidity Index. The primary outcomes of interest were the utilization trends of PE (treated with MT and ST); the secondary outcomes were mortality, discharge to facility, peri-procedural complications, and length of hospital (LOS) stay; the tertiary outcome was to identify the predictors of in-hospital mortality. From 2010–2018, there were 1,627,718 hospitalizations for PE, of which 6531 (0.39%) underwent MT and 3465 (0.21%) underwent ST. The utilization trend of MT increased from 336 (0.20%) in 2010 to 1655 (0.87%) in 2018; the utilization trend of ST was 260 (0.15%) in 2010 and 430 (0.23%) in 2018. The unadjusted in-hospital mortality for MT was 9.1% with the mean LOS being 7(±0.3) days; for ST, mortality was 13.9% with a mean LOS of 13(±0.4) days. The occurrences of periprocedural complications for MT and ST were as follows: invasive mechanical ventilation was 13.8% and 32%; cardiopulmonary bypass was 3.3% and 68.3%; pulmonary embolectomy surgery was 1.7%; and bleeding complications were 1.4% and 3.4%. Predictors associated with in-hospital mortality for MT were: increasing age (OR 1.2, 95% CI 1.0–1.3, *p* < 0.026), female sex (OR 1.9, 95% CI 1.2–2.8, *p* < 0.004), large hospitals (OR 2.2, 95% 1.4–3.5, *p* < 0.001), and teaching hospitals (OR 1.8, 95% CI 1.1–3.1, *p* < 0.023). The predictor of in-hospital mortality for ST was increasing age (OR 1.2, 95% CI 1.0–1.4, *p* < 0.046). The number of MT procedures performed has rapidly increased over the past decade. Further studies are warranted to determine their rise and therapeutic use.

## 1. Introduction

Pulmonary embolism (PE) is an acute and potentially fatal condition where embolic material, usually from a deep vein thrombus, blocks one or more pulmonary arteries resulting in impaired blood flow [1]. It is frequently underdiagnosed and the third most common cause of vascular diseases in the United States [2,3], with an annual prevalence of 66 cases per 100,000 population [4]. Massive and submassive PE can be treated with cardiopulmonary support, systemic anticoagulation, and thrombolysis [2,5]. PE treatments include interventions such as mechanical and surgical thrombectomy. Mechanical thrombectomy (MT) helps restore pulmonary circulation and avoid cardiogenic shock [6]. Surgical thrombectomy (ST) is advised for patients with rapid hemodynamic deterioration, clot-in-transit, or an underlying right-to-left shunt [7]. Catheter-directed thrombolysis has gained more popularity in recent times over anticoagulation alone as studies prove that it decreases RV/LV ratios [8]. Indications for mechanical thrombectomy include massive PE, failed thrombolysis, or contraindications to thrombolytic therapy [9]. Mechanical catheter-based thrombectomy may be the only choice for patients that are neither candidates for surgical embolectomy nor thrombolysis [10]. The CHEST Guideline and Expert Panel Report published in 2016 recommends mechanical thrombectomy for patients with acute PE and hypotension who have either high bleeding risk, failed systemic thrombolysis, or shock [11]. ST has a perioperative mortality rate of 27.2% [12], and is indicated in hemodynamically unstable patients with contraindication to thrombolysis, failed catheter therapy, or failed thrombolysis [13], amongst other contraindications. There are studies and case reports which discuss the advantages and disadvantages of mechanical thrombectomy and surgical thrombectomy, and also on the predictors of outcomes in pulmonary embolism, but there is limited information on the prevalence trends of MT and ST as well as on the predictors of outcomes [14]. We aim to describe the predictors of outcomes and complications of mechanical and surgical thrombectomy for pulmonary embolism by performing a population-based, retrospective cross-sectional study using national data. In this article, we studied the epidemiology of pulmonary embolism treated with mechanical and surgical embolectomy and their associated outcomes and complications.

## 2. Methods

### 2.1. Data Source

This is a retrospective study using data obtained from the Agency for Healthcare Research and Quality’s HCUP NIS files for 2010 to 2018. The NIS is the largest publicly available all-payer inpatient care database in the United States and contains discharge-level data provided by states that participate in it. This administrative dataset represents >95% of the national population. Each hospitalization is treated as an individual entry in the database and is coded with one principal diagnosis, up to 24 secondary diagnoses, and 15 procedural diagnoses associated with that stay. Detailed information on NIS is available at: www.hcup-us.ahrq. gov/db/nation/nis/nisdde.jsp (accessed on 30 September 2021). The NIS is a de-identified database and available to purchase after appropriate data use agreement and training, so informed consent or IRB approval was not needed for the study.

### 2.2. Study Population

We used the ninth and tenth revisions of the International Classification of Diseases (clinical modification codes ICD-9-CM and ICD-10-CM) to identify adult and pregnant patients admitted with a primary diagnosis of PE (ICD-10-CM codes I26.02, I26.09, I26.92, I26.93, I26.94, and I26.99; ICD-9-CM codes 415.11, 415.13, and 415.19). These codes have been previously validated and used in prior publications. Similarly, we used ICD-9-CM and ICD-10-CM codes to identify independent predictors, including comorbidities.

### 2.3. Definition of Data Elements

We extracted demographics, hospital-level characteristics (geographical region, size, and teaching status), and patient-level characteristics as supplied as part of NIS. Patient characteristics of interest were sex, age, race, insurance status, and concomitant diagnoses as defined above. The races were defined as white (referent), African American, Hispanic, Asian or Pacific Islander, and Native American. Insurance status was defined as being Medicare (referent), Medicaid, private insurance, or other/self-pay/no charge. We defined the severity of comorbid conditions using Deyo modification of the Elixhauser Comorbidity Index. HCUP NIS contains data on total charges for each hospital in the databases, which represent the amount that hospitals billed for services.

### 2.4. Outcomes

Our primary outcomes of interest were utilization trends of PE patients treated with mechanical thrombectomy and surgical thrombectomy and secondary outcomes were mortality, discharge to facility, peri-procedural complications, and length of hospital stay (LOS). The tertiary outcome was to identify the predictors of in-hospital mortality.

### 2.5. Statistical Analysis

To establish the trend, we calculated the utilization trends of MT and ST among PE hospitalizations. We used the Cochran–Armitage trend test to analyze the temporal trends. Descriptive statistics were performed to present the baseline difference in sociodemographic, comorbidities, and hospital-level characteristics by treatment type. Moreover, descriptive statistics were performed to outline post-processing outcomes. Categorical variables were compared with the chi-square test, and continuous variables were compared with the Student’s t-test or Wilcoxon rank-sum test. We used logistic regression models to identify the predictors of in-hospital mortality and adjusted them for potential confounders.

We utilized SAS 9.3 (SAS Institute, Cary, NC, USA) for all analyses and included designated weight values to produce nationally representative estimates. For regression models, we used survey procedures to account for the inherent survey design of NIS to produce more robust estimates. We considered a two-tailed *p*-value < 0.05 as statistically significant.

## 3. Results

### 3.1. Utilization Trend of Mechanical and Surgical Thrombectomy during Hospitalization Due to Pulmonary Embolism

**Figure 1:**
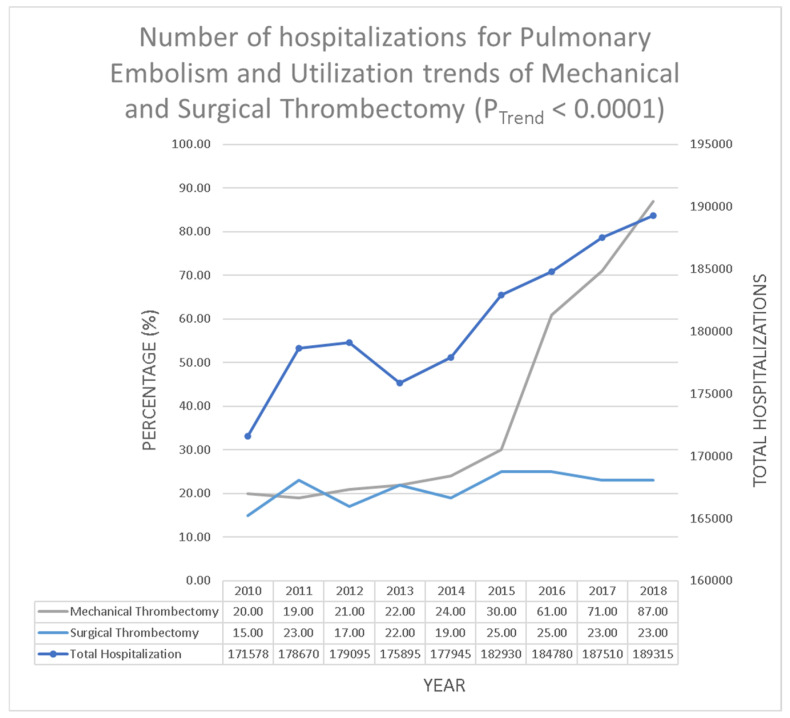
Number of hospitalizations for pulmonary embolism and utilization trends of mechanical and surgical thrombectomy (*P*_Trend_ < 0.0001).

In the years 2010–2018 there were a total 1,627,718 hospitalizations due to PE, out of which 6531 (0.39%) underwent MT and 3465 (0.21%) underwent ST. The utilization trend of MT significantly increased from 336 (0.20%) in 2010 to 1655 (0.87%) in 2018 (*p* < 0.01); however, the utilization trend of ST remained stable from 260 (0.15%) in 2010 to 430 (0.23%) in 2018 (*p* = 0.06) (Figure 1).

### 3.2. Baseline Characteristics of Pulmonary Embolism Patients Who Underwent Mechanical Thrombectomy

The majority of the MT patients were aged 65 years and older (45.1%), followed by those aged from 50–64 years old (31.6%). Both males and females were nearly equally represented in the MT population; males represented 50.8% of all MT patients while females made up 49.2%. White patients were represented the most at 66.5%, followed by Black patients at 18.8%. Patients with Medicare/Medicaid insurance also made up the majority of this group at 59.0%. Approximately 73.3% of the cases reported were admitted to teaching hospitals, and most patients were admitted via the emergency department (74.6%). The vast majority of patients presented with non-saddle type PE (69.4%), while saddle-type PE appeared in 30.6% of patients. About 93.8% of the mechanical thrombectomies performed were emergent. The most frequently occurring comorbid condition was hypertension which was seen in 60.5% of all patients receiving MT. Other commonly occurring comorbid conditions included fluid and electrolyte disorders (36.9%), obesity (29.8%), anemia (24.9%), and congestive heart failure (21.2%). The other baseline characteristics of this population are outlined in Table 1.

### 3.3. Outcomes and Peri-Procedural Complications of Pulmonary Embolism Patients Who Underwent Mechanical Thrombectomy

The overall unadjusted in-hospital mortality for patients undergoing mechanical thrombectomy (MT) was 9.1%, while 20.9% of patients were discharged to facilities and 70% of patients were discharged to home with the mean length of stay being 7(+/−0.3) days. Among the periprocedural complications, invasive mechanical ventilation (IMV) accounted for 13.8%, 3.3% patients underwent a cardiopulmonary bypass (CPB), 1.7% had to undergo pulmonary embolectomy surgery, and 1.4% had bleeding complications. Other observed complications are outlined in Table 2.

### 3.4. Baseline Characteristics of Pulmonary Embolism Patients Who Underwent Surgical Thrombectomy

The majority of hospitalized patients who underwent ST from 2010 to 2018 were between the ages of 50 and 64 years (34.8%), where the majority of the procedure was done in a large hospital bed (76.9%). Non-saddle pulmonary embolus was seen more than saddle pulmonary embolus (59.7% vs. 40.3%). More males than females underwent the procedure (53.8% vs. 46.2%) and White patients represented the largest ethnic group (65.8%), with Black patients representing the second largest ethnic group (18.2%). The most frequently occurring comorbid conditions were fluid and electrolyte disorders which were seen in 52.1% of all patients undergoing a surgical thrombectomy procedure. Other commonly occurring comorbid conditions were hypertension (50.8%), obesity (31.7%), congestive heart failure (30.1%), coagulopathy (29.3%), diabetes mellitus without chronic complications (15.5%), history of chronic pulmonary disease (15.2%), and valvular heart disease (12.1%). The majority of patients were insured under Medicare and Medicaid, with a percentage of 50.5%, while the percentage of uninsured/self-pay was 9.6%. The majority of patients came from the southern region (42.6%).

Emergency or urgent admissions accounted for 92.9%, with 79.8% of admissions occurring in teaching hospitals. The majority of patients’ source of admission was from the emergency department (56.5%). Other baseline characteristics are outlined in Table 1.

### 3.5. Outcomes and Peri-Procedural Complications of Pulmonary Embolism Patients Who Underwent Surgical Thrombectomy

The overall unadjusted in-hospital mortality for surgical thrombectomy (ST) was 13.9%, and discharge to other facilities was 32%. The mean length of stay following ST was 13 days (+/−0.4 days). The most common periprocedural complications associated with ST were cardiopulmonary bypass at 68.3%, invasive mechanical ventilation at 32%, cardiac arrest at 9.6%, ECMO at 3.7%, myocardial infarction at 3.4%, bleeding complications at 3.4%, and intracerebral hemorrhage at 0.9% (Table 2).

### 3.6. Predictors of In-Hospital Mortality of Pulmonary Embolism Patients Who Underwent Mechanical and Surgical Thrombectomy

From our analysis, several predictors were associated with an increased likelihood of in-hospital mortality among PE patients who underwent MT. These predictors include increasing age (OR 1.2, 95% CI 1.0–1.3, *p* < 0.026), female sex (OR 1.9, 95% CI 1.2–2.8, *p* < 0.004), large hospitals (OR 2.2, 95% 1.4–3.5, *p* < 0.001), and teaching hospitals (OR 1.8, 95% CI 1.1–3.1, *p* < 0.023).

Increasing age (OR 1.2, 95% CI 1.0–1.4, *p* < 0.046) was the only significant predictor for in-hospital mortality among patients with PE who underwent ST (Table 3 and Table 4).

## 4. Discussion

The Surgical Pulmonary Embolectomy As Routine therapy (SPEAR) working group reported 11.7% in-hospital postoperative mortality, of which 23.7% had massive PE and 9.1% had submassive PE [13]. Notably, 13.1% had CPR before ST with a significant difference between massive (34.2%) and submassive (8.5%) PE groups. Massive PE was associated with significantly worse postoperative outcomes including blood product transfusion (76.3% vs. 36.4% for submassive) and prolonged mechanical ventilatory requirement (42.1% vs. 25%). About 8.4% of patients required re-exploration for hemorrhage without a significant difference between the massive and the submassive PE groups. The SPEAR working group concluded that ST is a safe procedure at high-volume centers that can be used more frequently to treat patients presenting with an acute massive or submassive PE [15].

Current management of acute PE includes anticoagulation alone, systemic thrombolysis with anticoagulation, catheter-directed thrombolysis (CDT) with anticoagulation, or surgical thrombectomy [16].

Thrombolysis improves hemodynamics but is associated with bleeding complications. An ideal PE reperfusion strategy should be devoid of major complications. The goal of reperfusion is to improve systemic perfusion and survival and to prevent thromboembolic pulmonary hypertension. The choice of intervention is of significance and with varying rates of successes and complications. MT for the management of PE has risen markedly, despite the limitations on predictors of PE treatment outcomes. Such data would be invaluable in highlighting healthcare needs and also in academic research on PE. Currently, surgical thrombectomy is reserved for several specific clinical scenarios which generally include hemodynamic instability with concurrent inotropic or vasopressor support with contraindications for thrombolysis, failed thrombolysis with continued evidence of cardiopulmonary demise, and paradoxical clot-in-transit that is trapped within an atrial septal defect or patent foramen ovale [10].

The increase in the utilization trend of mechanical thrombectomy over the period of 2010 to 2018 may be attributed to the facilitation of the removal of thrombus, requirement of smaller doses of thrombolytic agents, shorter treatment times leading to improved relief of symptoms, decreased rate of complications, and more efficient patient care. Gayou et al. showed an increase in catheter-directed thrombolysis from 0.457 services to 5.057 services per 100,000 patients from 2004 to 2016 and the CDT-treated proportion of PE increased 10-fold from 2004 to 2016, which corresponds to an increase from 0.1% to 1.0% [17]. By contrast, the utilization trend of surgical thrombectomy remained relatively stable from 2010 to 2018 due to the specific requirements needed for its use, namely being cases of large thrombus, failed catheter therapy, or thrombolysis or contraindications to thrombolysis. These requirements in themselves lower the proportion of the population treated with surgical thrombectomy. Moreover, surgical thrombectomy has risks of excessive bleeding that can be severe enough to cause death, mechanical damage to blood vessels and nearby organs at the surgical site of a blood clot, infection, and reaction to anesthesia. However, one study comprising of 23,858 patients with PE from 2001 to 2013 showed an increase in the use of surgical thrombectomy from 0.3% to 0.6% [18]. Another study of 1,916,793 patients with PE also showed similar results of significant increase in the use of surgical thrombectomy over the period of 2003 to 2014; this increase may be due to patients’ critically ill status at baseline, advanced disease, or the failure of thrombolytic therapy [19].

Studies have reported varied mortality rates for ST. Whilst treatment options are growing, there are controversies surrounding the appropriate utility of these advanced therapies in different clinical settings.

Mortality post ST has been on the rise between 2010 and 2018, owing to patient-related or procedure-related complications. Furthermore, as the population continues to age, the incidence of pulmonary embolic events will escalate, as increasing age is a significant predictor of mortality.

CDT may be the only feasible approach for high-risk PE and for patients not suitable for thrombolysis or ST [10].

A retrospective study based on data collected from 1998 to 2014 from the Society of Thoracic Surgeons showed an overall mortality rate of 11.7% amongst patients who underwent ST. Patients who had massive PE had a mortality rate of 23.7%, while the submassive PE population experienced a mortality rate of 9.1% [13].

Our study found that old age was a significant predictor of inpatient mortality in PE patients that underwent either MT or ST. The published literature is scarce on increasing age as a predictor of mortality among treated PE patients; however, there are publications that demonstrated increasing age was associated with worse outcomes in PE patients [12]. Kilic et al. showed that in patients who underwent surgical embolectomy, Black race was associated with greater in-patient mortality compared to White race [12]. Another study found in their analysis of the NIS that the most significant predictor of in-hospital mortality was the use of thrombolytics before surgical thrombectomy in PE patients [19]. Additionally, our results demonstrated an increased risk of in-hospital death with female sex (OR 1.9), large beds (OR 2.2) and teaching hospitals (OR 1.8). This could be due to a higher influx of more ill and complex PE cases to these hospitals as opposed to smaller community hospitals that may lack the facilities and expertise required for mechanical and surgical thrombectomy. There is a lack in the literature of support for any sex differences in the mortality outcomes of PE patients treated with either surgical or mechanical thrombectomy.

The treatment options involving catheter-based therapies and surgical thrombectomy are constantly evolving especially for the intermediate and high-risk categories of patients in whom thrombolysis is contraindicated. In particular, the drawback of surgical therapy for massive or submassive PE in patients in whom thrombolysis therapy or mechanical thrombectomy is absolutely contraindicated or has failed appears to be too restrictive. There are no current specific guidelines which focus on the favorable clinical and functional outcomes of surgical thrombectomy. There is a need for multicenter studies to be conducted and national registries should be maintained to provide missing data in order to improve patient allocation and treatment algorithms. For this reason, our study focused on the utilization trends of surgical thrombectomy and the associated demographic details and various complications seen post-surgery, which can inspire further studies in this regard to better understand the reasons for implementation of this procedure in future treatment protocol.

## 5. Limitations

The study results are limited by the retrospective analysis of data that may be affected by missing data or administrative erroneous coding inputs. Given that ICD-9 and ICD-10 codes were used, PE coding diagnosis and description error may be assumed. However, the size of the dataset used for the analysis is expected to offset any errors associated with administrative recording, which would not interfere with the final conclusion of the study. Additionally, the authors cannot differentiate for ST whether cardiopulmonary bypass was a complication of the treatment or a routine part of the surgery, which may render the resulting number an inaccurate reflection of the complication.

## 6. Conclusions

In the past decade, pulmonary embolism hospitalizations have steadily and slowly increased. Pharmacological treatment and therapeutic procedures for the management of pulmonary embolism are constantly advancing. In recent years, we found that there has been a drastic rise in using mechanical thrombectomy to treat patients hospitalized with pulmonary embolism, without significant increase in related complications, while surgical thrombectomy use has largely remained approximately stable. The implications of the rise in mechanical thrombectomy, as well as its safety and efficacy, warrant further detailed studies.

## Figures and Tables

**Table 1:** Baseline characteristics of inpatient population undergoing mechanical thrombectomy and surgical thrombectomy.

Characteristics	Mechanical Thrombectomy	%	Surgical Thrombectomy	%
**Overall**	6531	100	3465	100
**Age in years (%)**				
18–34	425	6.5	414	11.9
35–49	1093	16.7	722	20.8
50–64	2065	31.6	1204	34.8
≥65	2947	45.1	1126	32.5
**Sex (%)**				
Male	3320	50.8	1866	53.8
Female	3211	49.2	1599	46.2
**Race (%)**				
White	4343	66.5	2280	65.8
Black	1231	18.8	629	18.2
Hispanic	388	5.9	195	5.6
Others	253	3.9	173	5.0
Missing	317	4.9	187	5.4
**Comorbidities (%)**				
Obesity	1947	29.8	1097	31.7
Hypertension	3953	60.5	1761	50.8
Diabetes mellitus with chronic complications	628	9.6	218	6.3
Diabetes mellitus without chronic complications	1073	16.4	538	15.5
Congestive heart failure	1387	21.2	1042	30.1
Valvular heart disease	446	6.8	420	12.1
History of chronic pulmonary disease	1249	19.1	526	15.2
Peripheral vascular disease	621	9.5	213	6.2
Coagulopathy	1131	17.3	1015	29.3
Solid tumor without metastasis	324	5.0	112	3.2
Metastatic cancer	352	5.4	100	2.9
Liver disorders	239	3.7	89	2.6
Weight loss	364	5.6	364	10.5
Alcoholism	239	3.7	148	4.3
Other neurological disorders	482	7.4	304	8.8
Renal failure	924	14.1	263	7.6
Hypothyroidism	727	11.1	341	9.8
Anemia	1624	24.9	601	17.4
Fluid and electrolyte disorders	2407	36.9	1804	52.1
Depression	703	10.8	365	10.5
**Type of PE (%)**				
**Non-saddle**	4531	69.4	2068	59.7
**Saddle**	1999	30.6	1397	40.3
**Median household income (%)**				
1st quartile	1919	29.4	931	26.9
2nd quartile	1607	24.6	831	24.0
3rd quartile	1604	24.6	852	24.6
4th quartile	1301	19.9	767	22.1
**Primary insurance (%)**				
Medicare/Medicaid	3856	59.0	1749	50.5
Private including HMO	2233	34.2	1384	39.9
Uninsured/self-pay	427	6.5	333	9.6
**Hospital bed size (%)**				
Small	695	10.6	198	5.7
Medium	1567	24.0	598	17.3
Large	4261	65.2	2664	76.9
**Hospital type (%)**				
Rural	277	4.2	59	1.7
Urban nonteaching	1461	22.4	637	18.4
Teaching	4784	73.3	2764	79.8
**Hospital region (%)**				
Northeast	981	15.0	792	22.9
Midwest	1616	24.7	756	21.8
South	2553	39.1	1477	42.6
West	1382	21.2	440	12.7
**Day of admission**				
Weekday	5089	77.9	2621	75.6
Weekend	1442	22.1	844	24.4
**Source of admission (%)**				
Transfer from other hospital or other health facility	1659	25.4	1508	43.5
Emergency department	4872	74.6	1956	56.5
**Type of admission (%)**				
Emergent or urgent	6090	93.8	3208	92.9
Elective	405	6.2	247	7.1

**Table 2:** Complications of mechanical thrombectomy and surgical thrombectomy.

Complications	Mechanical Thrombectomy	%	Surgical Thrombectomy	%
In-hospital mortality	592	9.1	482	13.9
Discharge to facility	1365	20.9	1108	32.0
Length of stay (days)	7	0.3	13	0.4
Cardiopulmonary bypass	217	3.3	2365	68.3
ECMO	35	0.5	130	3.7
Converted to surgical thrombectomy	113	1.7	-	-
Invasive mechanical ventilation	902	13.8	1108	32.0
Intracerebral hemorrhage	59	0.9	30	0.9
Cardiac arrest	152	2.3	333	9.6
Myocardial infarction	710	10.9	117	3.4
Bleeding complications	93	1.4	118	3.4

**Table 3:** Predictors of mortality in mechanical thrombectomy.

Independent Variable/Characteristic	Odd Ratio	95% CI (LL)	95% CI (UL)	*p*-Value
**Age (10 years increase)**	1.2	1.0	1.3	0.026
**Sex**				
Female vs. male	1.9	1.2	2.8	0.004
**Race**				
Non-white vs. white	0.9	0.6	1.4	0.570
**Elixhauser comorbidity score (10-point increment)**	1.0	0.9	1.1	0.792
**Hospital bed size**				
Large vs. small/medium	2.2	1.4	3.5	0.001
**Hospital type**				
Teaching vs. rural/urban nonteaching	1.8	1.1	3.1	0.023
**Hospital region**				
Northeast vs. west	1.0	0.5	1.9	0.964
Midwest vs. west	0.9	0.5	1.6	0.676
South vs. west	0.8	0.4	1.3	0.335
**Day of admission**				
Weekend vs. weekday	0.9	0.6	1.5	0.784
**Source of admission (%)**				
Transfer from other hospital or other health facility vs. emergency department	1.2	0.7	1.9	0.483
**Type of admission (%)**				
Elective vs. emergent or urgent	0.4	0.1	1.5	0.193

**Table 4:** Predictors of mortality in surgical thrombectomy.

Independent Variable/Characteristic	Odd Ratio	95% CI (LL)	95% CI (UL)	*p*-Value
**Age (10 years increase)**	1.2	1.0	1.4	0.046
**Sex**				
Female vs. male	1.2	0.8	1.9	0.343
**Race**				
Non-white vs. white	0.8	0.5	1.3	0.373
**Elixhauser comorbidity score (10-point increment)**	1.0	0.9	1.0	0.577
**Hospital bed size**				
Large vs. small/medium	0.8	0.5	1.2	0.259
**Hospital type**				
Teaching vs. rural/urban nonteaching	0.9	0.6	1.5	0.686
**Hospital region**				
Northeast vs. west	1.0	0.4	2.2	0.987
Midwest vs. west	0.9	0.4	2.1	0.888
South vs. west	1.0	0.5	2.2	0.926
**Day of admission**				
Weekend vs. weekday	0.7	0.4	1.2	0.168
**Source of admission (%)**				
Transfer from other hospital or other health facility vs. emergency department	0.6	0.4	1.1	0.086
**Type of admission (%)**				
Elective vs. emergent or urgent	0.7	0.3	1.8	0.453

## Data Availability

The Nationwide Inpatient Sample (NIS) data that support the findings of this study are openly available from the Healthcare Cost and Utilization Project (HCUP) at https://www.hcup-us.ahrq.gov/db/nation/nis/nisdbdocumentation.jsp (accessed on 30 September 2021).
